# Ginsenoside Rh2 regulates triple-negative breast cancer proliferation and apoptosis via the IL-6/JAK2/STAT3 pathway

**DOI:** 10.3389/fphar.2024.1483896

**Published:** 2025-01-08

**Authors:** Rumeng Ding, Quancheng Kan, Ting Wang, Ran Xiao, Yanan Song, Duolu Li

**Affiliations:** Department of Pharmacy, The First Affiliated Hospital of Zhengzhou University, Zhengzhou, Henan, China

**Keywords:** ginsenoside Rh2, triple-negative breast cancer, network Pharmacology, IL-6/JAK2/STAT3 pathway, apoptosis

## Abstract

**Introduction:**

Triple-negative breast cancer (TNBC) is the most challenging subtype of breast cancer to treat. While previous studies have demonstrated that ginsenoside Rh2 induces apoptosis in TNBC cells, the specific molecular targets and underlying mechanisms remain poorly understood. This study aims to uncover the molecular mechanisms through which ginsenoside Rh2 regulates apoptosis and proliferation in TNBC, offering new insights into its therapeutic potential.

**Methods:**

Network analysis and transcriptome sequencing were utilized to explore the potential mechanisms of ginsenoside Rh2 in treating TNBC. *In vivo* imaging and immunohistochemistry were employed to examine the effects of ginsenoside Rh2 in a TNBC mouse model. Functional assays were conducted to assess the impact of ginsenoside Rh2 on TNBC cell behavior. Additionally, ELISA, Western blot, and quantitative real-time PCR were used to further investigate the mechanisms of ginsenoside Rh2-induced apoptosis in TNBC cells.

**Results:**

Through network analysis, 47 common targets were identified, and Gene Ontology (GO) enrichment analysis suggested that ginsenoside Rh2 may exert therapeutic effects in TNBC by influencing apoptosis, cell proliferation, and protein kinase activity. Both transcriptomic analysis and network analysis revealed the JAK/STAT signaling pathway as a key mechanism. Ginsenoside Rh2 inhibited tumor growth in TNBC mice and reduced the expression of IL- 6, IL-6R, STAT3, Bcl-2, and Bcl-xL in tumor tissues. The ability of ginsenoside Rh2 to inhibit TNBC cell proliferation was further confirmed by attenuating the activation of the IL-6/JAK2/STAT3 apoptosis pathway and reducing the expression of protein kinases AMPK-α1 and PKA-Cα.

**Conclusion:**

Based on network analysis and experimental validation, our findings demonstrate that ginsenoside Rh2 regulates TNBC proliferation and apoptosis through suppression of the IL-6/JAK2/STAT3 pathway, both *in vitro* and *in vivo*. This comprehensive approach represents a significant advancement in understanding the therapeutic potential of ginsenoside Rh2 in treating TNBC.

## 1 Introduction

According to the most recent global cancer statistics, breast cancer has now surpassed lung cancer as the most prevalent malignancy worldwide (Siegel et al., 2023). Triple-negative breast cancer (TNBC), defined by the absence of estrogen receptor (ER), progesterone receptor (PR), and human epidermal growth factor receptor 2 (HER2), represents the most aggressive subtype of breast cancer (Gajulapalli et al., 2016). Accounting for 10%–15% of all breast cancer cases, TNBC is characterized by its highly malignant nature and poor prognosis, making it particularly difficult to treat (de Ruijter et al., 2011; Howlader et al., 2017; Garrido-Castro et al., 2019; Siegel et al., 2019; Yin et al., 2020). Its high invasiveness and the lack of effective molecular targets have led chemotherapy to remain the primary treatment modality for advanced and unresectable TNBC. However, the clinical efficacy of chemotherapy is often limited by the development of drug resistance (Dent et al., 2007; Bianchini et al., 2016; Bianchini et al., 2022). Recent advancements in targeted therapies and immune checkpoint inhibitors have been incorporated into clinical practice, showing promising results for some advanced TNBC patients (Lyons, 2019). However, these therapies benefit only a subset of patients in clinical settings (Dirix et al., 2018; Schmid et al., 2018). Therefore, there is an urgent need to discover novel, safe, and effective therapeutic agents and to better understand the underlying molecular mechanisms driving TNBC.

*Panax ginseng* C.A. Mey, a member of the Araliaceae family, is renowned for its tonifying effects, traditionally used to treat various deficiency syndromes. Ginsenoside Rh2, a steroidal saponin isolated from Panax ginseng, consists of two optical isomers, 20(S)-Rh2 and 20(R)-Rh2, with the former demonstrating superior anti-tumor activity (Li et al., 2020; Zeng et al., 2020). Ginsenoside Rh2 is recognized for its low toxicity and has been investigated for its potential in tumor prevention and therapy across various cancers. Notably, 20(S)-Rh2 has been shown to inhibit the proliferation of malignant tumors, induce cell cycle arrest, and promote apoptosis in cancers such as liver, lung, melanoma, leukemia, colon, ovarian, and breast cancer (Li et al., 2017; Xia et al., 2017; Gao and Zheng, 2018; Zhang et al., 2021; Zhang et al., 2022). Additionally, ginsenoside Rh2 has been reported to mitigate chemotherapy- and radiotherapy-induced side effects, including nausea, vomiting, hair loss, anemia, and anorexia (Jia et al., 2004). Studies have also demonstrated that ginsenoside Rh2 inhibits TNBC metastasis by targeting the EGFR-mediated MAPK signaling pathway (Peng et al., 2019). However, the effects of 20(S)-Rh2 on TNBC apoptosis remain underexplored, and the specific molecular mechanisms involved are not fully understood.

In this study, we employed network analysis and RNA-seq to predict the potential molecular targets, biological processes, and mechanisms through which ginsenoside Rh2 may regulate TNBC proliferation and apoptosis. These findings were subsequently validated through both *in vitro* and *in vivo* experiments. Our research aims to uncover novel mechanistic insights and provide promising new approaches for the development of therapeutic strategies targeting TNBC.

## 2 Materials and methods

### 2.1 Network analysis

The PubChem database (https://pubchem.ncbi.nlm.nih.gov/) was used to explore SMILES of ginsenoside Rh2, and the SMILES was input to the Swiss Target Prediction (http://www.swisstargetprediction.ch/) to obtain potential targets of ginsenoside Rh2. The keyword ‘triple-negative breast cancer’ was entered into three databases, DisGeNET (https://www.disgenet.org/), GeneCards (https://www.genecards.org/) and OMIM (https://www.omim.org/) respectively, to searching for disease target genes. A Venn diagram was used to show the common targets of ginsenoside Rh2 and TNBC. We utilized the UniProt (https://www.uniprot.org/) database to perform name normalization of the target molecules. The common targets were input into the STRING database (https://cn.string-db.org/) to obtain protein-protein interaction (PPI) information, and then the Cytoscape 3.10.1 software was applied to draw the PPI network diagram. The plug-in, CentiScape 2.2 was used to further select core targets by the values of ‘Degree’, ‘Closeness’, and ‘Betweenness’. The core genes were imported into the DAVID database (https://david.ncifcrf.gov/summary.jsp) for GO and Kyoto Encyclopedia of Genes and Genomes (KEGG) enrichment analysis, and the relevant information of biological process (BP), cellular component (CC), molecular function (MF), KEGG pathways was acquired. Bioinformatics (https://www.bioinformatics.com.cn/) was applied to visualize the top 10 GO terms and the top 20 KEGG pathways with maximum enrichment.

### 2.2 Construction of tumor-bearing mouse model

Female Balb/c mice (n = 10, 6 weeks old) were purchased from the Beijing Vital River Laboratory Animal Technology Co., Ltd., license number: SCXK (Beijing) 2021–0006. The animals were randomly assigned into two groups: control and ginsenoside Rh2 (MedChemExpress, Monmouth Junction, NJ, United States) treated group. The purity of ginsenoside Rh2 was 98.11%. Each group contained five mice. Luciferase-labelled 4T1 cell suspension was injected subcutaneously at a density of 1 × 10^6^ cells into the flank of mice in control and ginsenoside Rh2 groups. A week after the 4T1 cell injection, the mice were intraperitoneally injected every 2 days with ginsenoside Rh2 (50 mg/kg) or saline (KELUN, Henan, China) for 3 weeks (Lev-Ari et al., 2021; Hou et al., 2022). At the end of administration of 20(S)-Rh2, mice were intraperitoneally injected with luciferin (15 mg/mL) at a dosage of 10 μL/g. Subsequently, *in vivo* imaging was conducted within 30 min, followed by the analysis of bioluminescence in tumor tissues. After the animal experiment, euthanasia was performed on the mice. A midline incision was made to access the tumor site, and tumor tissues were carefully excised. The excised tissues were immediately placed in liquid nitrogen and 4% paraformaldehyde to preserve their integrity. This experiment has been approved by the laboratory animal ethics committee of the First Affiliated Hospital of Zhengzhou University (Ethics Approval No.: 2022-KY-0001–002).

### 2.3 UID RNA-seq experiment

Total RNA was isolated from tumor mass using TRIzol Reagent (Invitrogen). With NanoDrop (Thermo Scientific, Waltham, Mass, United States), we further assessed the RNA concentration and purity. The RNA integrity was confirmed by 1.5% agarose gel electrophoresis and finally quantified by Qubit3.0 with Qubit™ RNA Broad Range Assay kit (Invitrogen). Subsequently, the 2 μg total RNA samples were used for RNA sequencing by processing with KC-Digital™ Stranded mRNA Library Prep Kit for Illumina^®^ (Wuhan Seqhealth Co., Ltd. Wuhan, China).

### 2.4 Immunohistochemistry (IHC) staining

Paraffin sections of TNBC mouse tumor tissues fixed with 4% paraformaldehyde were prepared. Paraffin sections dewaxed and hydrated. The tissue sections were heated in citric acid antigen repair buffer (PH6.0) for 10 min at 100 °C for antigen repair. The section was put into a 3% hydrogen peroxide solution to block endogenous peroxidase. The tissue was uniformly covered with 3%BSA and closed at room temperature for 30 min. Add a certain proportion of diluted primary antibody and incubate at 4 °C overnight. After adding the second antibody, color development, microscopic examination, and image acquisition analysis were carried out. The dilution ratio of primary antibody is as follows: anti-IL-6 (1:200, abs135607, absin, Shanghai, China), IL-6R (1:200, 23457-1-AP, Proteintech Group, Wuhan, China), STAT3 (1:200, 30835S, CST, Danvers, MA, United States), Bcl-2 (1:300, ab182858, abcam, Cambridge, England), and Bcl-xL (1:500, #2764, CST).

### 2.5 Cell and culture

The human mammary epithelial cell line, HBL-100, TNBC cell lines, MDA-MB-231, MDA-MB-468, and the mice breast cancer cell line (4T1) were purchased from the American Type Culture Collection (ATCC; Manassas, VA, United States). The cell lines were incubated in RPMI-1640 (Solarbio, Beijing, China) culture medium containing 10% fetal bovine serum (FBS) (Invitrogen, Carlsbad, CA, United States) at 37°C and 5% CO_2_.

### 2.6 Cell viability assay

Cell viability of TNBC cells was performed with Cell Counting Kit-8 (CCK-8; Kemix, Beijing, China), which was used to evaluate the growth inhibition induced by increasing concentrations of ginsenoside Rh2. Briefly, we seeded exponentially growing HBL-100 and TNBC cells into a 96-well plate at 6 × 10^3^ cells/well. The cells were processed by gradually varying concentrations of ginsenoside Rh2 for 48 h. Subsequently, 10 μL of CCK8 reagent was added. The absorbance was analyzed at 450 nm utilizing the 96-well SpectraMax plate reader after a further incubation period of 2 h.

### 2.7 Clone formation experiment

2 mL complete medium including various concentrations of ginsenoside Rh2 mixed with 500 cells was added to each well of a 6-well plate. The 6-well plate was placed in the incubator for further cultivation. After 10 days, paraformaldehyde (biosharp, Beijing, China) was fixed overnight and 0.1% crystal violet (meilunbio, Dalian, China) was dyed for 1 h. The number of clones was assessed by photographing after water washing.

### 2.8 Transwell migration and invasion assay

8-mm Pore Transwell Inserts (BIOFIL, Guangzhou, China) was used in the transwell chambers to analyze the migratory and invasive capacity of TNBC cells. Samples each containing 2.5 × 10^4^ cells in 100 μL RPMI-1640 with 2% FBS were added. The Matrigel (Corning, New York, United States) was not covered above the chamber when testing the migration ability of TNBC cells, while it was needed when testing the invasion ability. Then the lower chamber added 10% FBS (600 μL). Cells on Transwell Inserts were fixed with paraformaldehyde overnight. The cells were dyed evenly by utilizing 0.1% crystal violet. Cells passing through the chamber were visualized utilizing an electron microscope, followed by quantification. The migration rate was expressed as the number of migrated cells.

### 2.9 Apoptosis analysis

By utilizing the method of Hoechst 33258 staining and flow cytometry, we assessed TNBC cell apoptosis with ginsenoside Rh2 treatment. For Hoechst staining, cells were planted into a 6-well plate including a cover slip. The TNBC cells were treated with ginsenoside 20(S)-Rh2 after 24 h of cell adhesion. Cells were cultured for 24 h and then were fixed with paraformaldehyde. And staining was performed with Hoechst 33,258 (Beyotime, Shanghai, China) for 5 min. After using an anti-fluorescence quenching liquid (Beyoyime), the state of the cell nucleus was observed under the fluorescence microscope. In the experimental assessment of apoptosis in TNBC cells using flow cytometry, we employed the FITC and Annexin V reagents (BD, NJ, United States, #556547) to estimate the apoptosis rate of TNBC cells, following the manufacturer′s instructions.

### 2.10 Quantitative real-time PCR

Total RNA was isolated from the Rh2-treated TNBC cells by utilizing the RNA Extraction kit. Then, we applied the Reverse Transcription System kit (Takara, Japan) to create the cDNA. Subsequently, we further quantified the expression of mRNAs by RT-PCR with SYBR Green (Takara). GAPDH, commonly known as the housekeeping gene, was utilized as an internal control. Genes and their corresponding specific primers (5′-3′) are shown in Table 1.

**TABLE 1:** List of PCR primers.

Gene name	Primer sequence (5′–3′)
GAPDH Forward	GAC​CAC​AGT​CCA​TGC​CAT​CAC
GAPDH Reverse	GTC​CAC​CAC​CCT​GTT​GCT​GTA
Bcl-2 Forward	GGT​GGG​GTC​ATG​TGT​GTG​G
Bcl-2 Reverse	CGG​TTC​AGG​TAC​TCA​GTC​ATC​C
Bcl-xL Forward	GAG​CTG​GTG​GTT​GAC​TTT​CTC
Bcl-xL Reverse	TCC​ATC​TCC​GAT​TCA​GTC​CCT
PKA-Cα Forward	TAC​CCG​CCC​TTC​TTC​GCA​GAC
PKA-Cα Reverse	GTT​CCG​CAG​CAG​GTC​CTT​CAA​G
AMPK-α1 Forward	AGA​AAG​TCG​GCG​TCT​GTT​CCA​AC
AMPK-α1 Reverse	CAT​TCA​TGT​GTG​CAT​CAA​GCA​GGA​C

### 2.11 Western blot

The TNBC cells on a 6-well plate were treated with different concentrations of ginsenoside Rh2 and lysed by strong RIPA lysis buffer (CWBIO, Jiangsu, China). Total proteins were collected after centrifugation at 13,000 rpm for 6 min. By using a BCA kit (Solarbio), the total protein concentration was quantified following the instructions. Subsequently, the proteins were denatured at 98°C for 10 min, and 30 μg of the protein sample was separated using 4%–20% sodium dodecyl sulfate-polyacrylamide gel electrophoresis. Proteins were transferred onto the polyvinylidene fluoride (PVDF) membrane (Millipore, NY, United States) and then the protein-free rapid blocking buffer (Epizyme, Shanghai, China) was used for blocking the membrane for 20 min. Afterward, tris-buffered saline containing Tween-20 (TBST; ZHONGHUIHECAI, Shanxi, China) was used to wash the PVDF membrane. The membrane was incubated overnight by rabbit mAb primary antibodies used are as follows: Bcl-2 (1:1,000, #3498, CST); Bcl-xL (1:1,000, #2764, CST); BAX (1:1,000, #14796, CST), Stat3 (1:1,000, #30835, CST); p-Stat3 (1:1,000, #94994, CST); Jak2 (1:1,000, #3230, CST); p-Jak2 (1:1,000, #4406, CST); IL-6 (1:1,000, ab259341, abcam); IL-6R (1:1,000, ab271042, abcam); AMPK α1 (1:1,000, ab32047, abcam) PKA-Cα (1:1,000, #5842, CST). Subsequently, the blots were incubated with goat anti-mouse (1:10,000, abs20039, absin) or anti-rabbit IgG-HPR (1:10,000, abs20040, absin), followed by determination with ECL Western Blotting Substrate (Solarbio). GAPDH (1:10,000, #AF7021, Affinity, Jiangsu, China) was used as the loading control.

### 2.12 ELISA assay

TNBC cells were seeded into six-well plates, and after cell adherence, they were treated with ginsenoside Rh2 (40 μM) for 36 h. Subsequently, the cell culture supernatant was collected, and the secretion of IL-6 by TNBC cells was assessed using the BD OptEIA™ Human IL-6 ELISA Set (BD, Cat. #555220) in combination with the BD OptEIA™ Reagent Set B (BD, Cat. #550534) following the manufacturer′s instructions.

### 2.13 Statistical analyses

Statistical analyses were carried out using Prism 8.0 (GraphPad). Data were acquired from at least three independent repeated experiments. Unpaired Student's *t*-test and One-way analysis of variance were used for analyzing statistical significance. A *P*-value <0.05 was considered significant.

## 3 Results

### 3.1 Network analysis prediction

**FIGURE 1:**
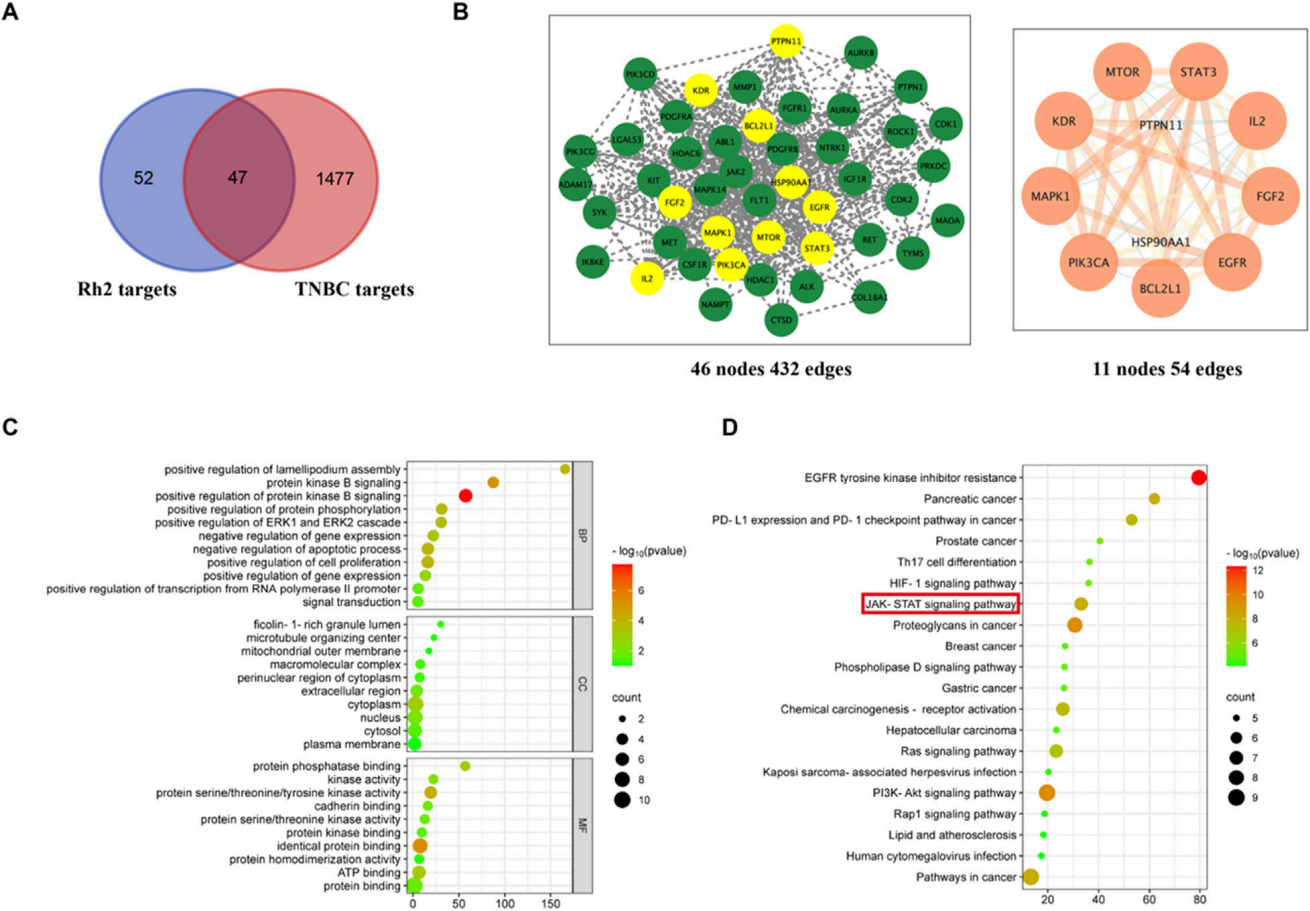
Network analysis prediction. **(A)** Venn diagram of common targets of ginsenoside and TNBC. **(B)** PPI network diagrams of common targets and core targets. **(C)** GO enrichment. **(D)** KEGG enrichment.

99 targets of ginsenoside Rh2 were obtained through the Swiss Target Prediction database. 151 disease targets and 511 disease targets were found in DisGeNET and OMIM databases respectively, and 1,034 gene targets were screened in the GeneCards database according to the median value of relevance score. Disease targets obtained from the above three databases were combined, and then duplicates were removed. A total of 1,524 disease targets were acquired. As shown in the Venn diagram, 47 common targets of ginsenoside Rh2 and disease targets were intersected (Figure 1A). The common targets were input into the STRING database and the PPI network was constructed using Cytoscape. CentiScape 2.2 plug-in was applied to analyze the PPI network to acquire core targets. The following are the 11 core targets: STAT3, EGFR, BCL2L1, PIK3CA, MAPK1, KDR, MTOR, IL-2, FGF2, PTPN11, HSP90AA1 (Figure 1B). The results of GO enrichment analysis showed that the primary biological processes are protein kinase B signaling, negative regulation of the apoptotic process, positive regulation of cell proliferation, etc., the primary cellular components are cytoplasm, nucleus, plasma membrane, etc., and the main molecular functions are identical protein binding, protein kinase activity, ATP binding, etc (Figure 1C). The molecular mechanisms may be associated with the JAK-STAT signaling pathway, PD-L1 expression and PD-1 checkpoint pathway, PI3K-AKT, Rap1, HIF signaling pathway, and EGFR tyrosine kinase inhibitor resistance (Figure 1D).

### 3.2 RNA-seq analysis of TNBC mouse tumor tissue treated with ginsenoside Rh2

**FIGURE 2:**
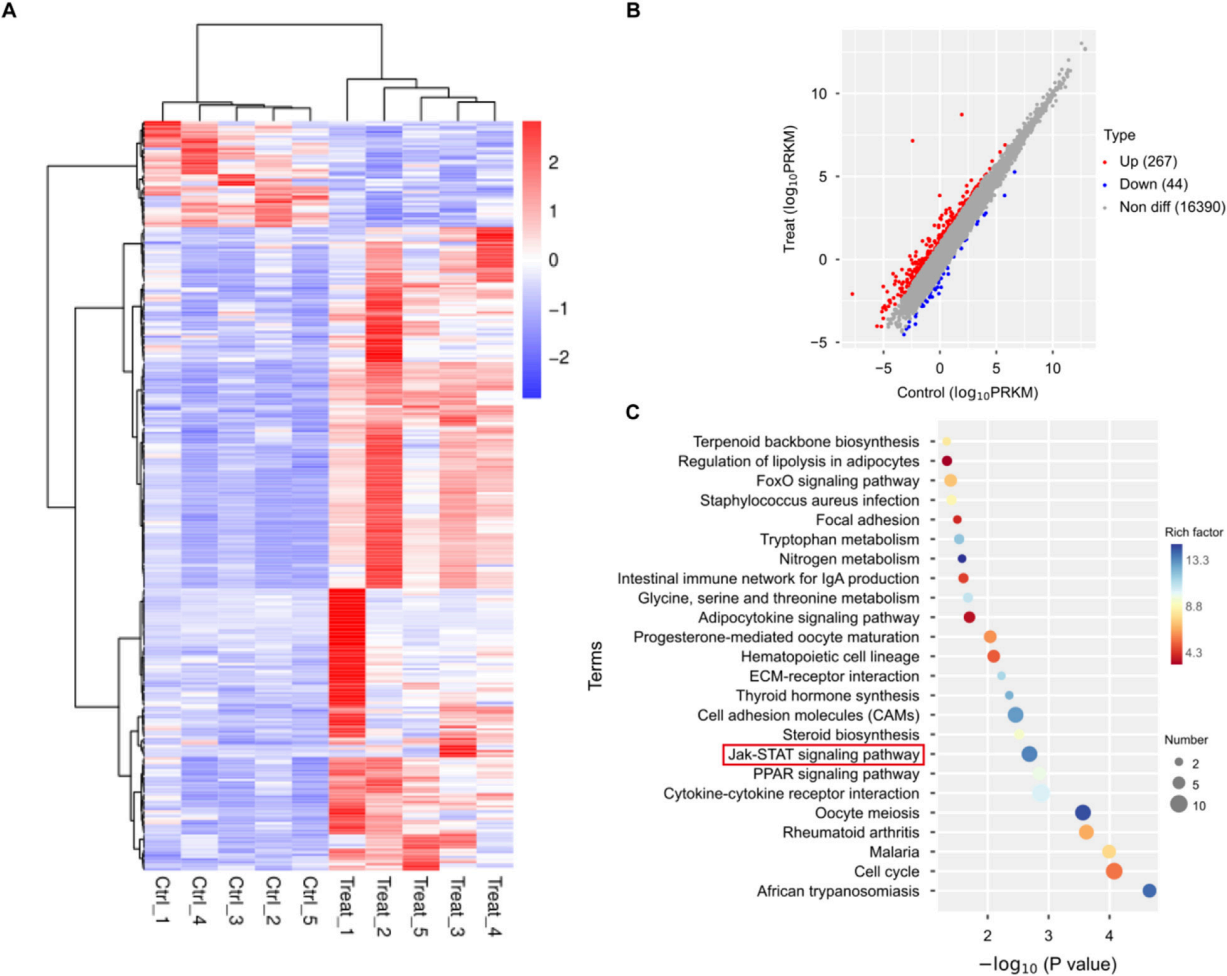
Transcriptome analysis of TNBC mouse tumor tissue treated with ginsenoside Rh2. **(A)** Heatmap of differentially expressed genes. **(B)** Both upregulated and downregulated genes in the ginsenoside Rh2-treated mouse model compared to the control group. **(C)** KEGG pathway enrichment analysis with differentially expressed genes.

To further explore the molecular mechanisms of 20(S)-Rh2 on TNBC, we collected tumor tissue from ginsenoside Rh2-treated and control groups of TNBC-bearing mice. Transcriptional profiling was performed using the Illumina sequencing platform. Through transcriptome sequencing analysis, a total of 311 differentially expressed genes were identified, comprising 267 upregulated and 44 downregulated genes (Figures 2A, B). Subsequently, KEGG pathway enrichment analysis of these differentially expressed genes revealed 24 enriched signaling pathways (Figure 2C). Combining network analysis predictions and sequencing results, we noted the JAK/STAT signaling pathway. It is worth noting that several studies have indicated that STAT3, a member of the STAT family, is an oncogene and is aberrantly activated in various malignant tumors, including breast cancer (Yu et al., 2014; Guanizo et al., 2018). And the inhibition of STAT3 expression significantly suppresses tumor growth and proliferation. Additionally, the literature suggests that the IL-6/JAK2/STAT3 signaling pathway is an upstream regulator of anti-apoptotic genes such as Bcl-2 and Bcl-xL. Therefore, we hypothesize that the IL-6/JAK2/STAT3 signaling pathway may regulate the apoptosis and proliferation of TNBC and verify this mechanism through *in vivo* and *in vitro* experiments.

### 3.3 Ginsenoside Rh2 can protect against tumor growth in TNBC models

**FIGURE 3:**
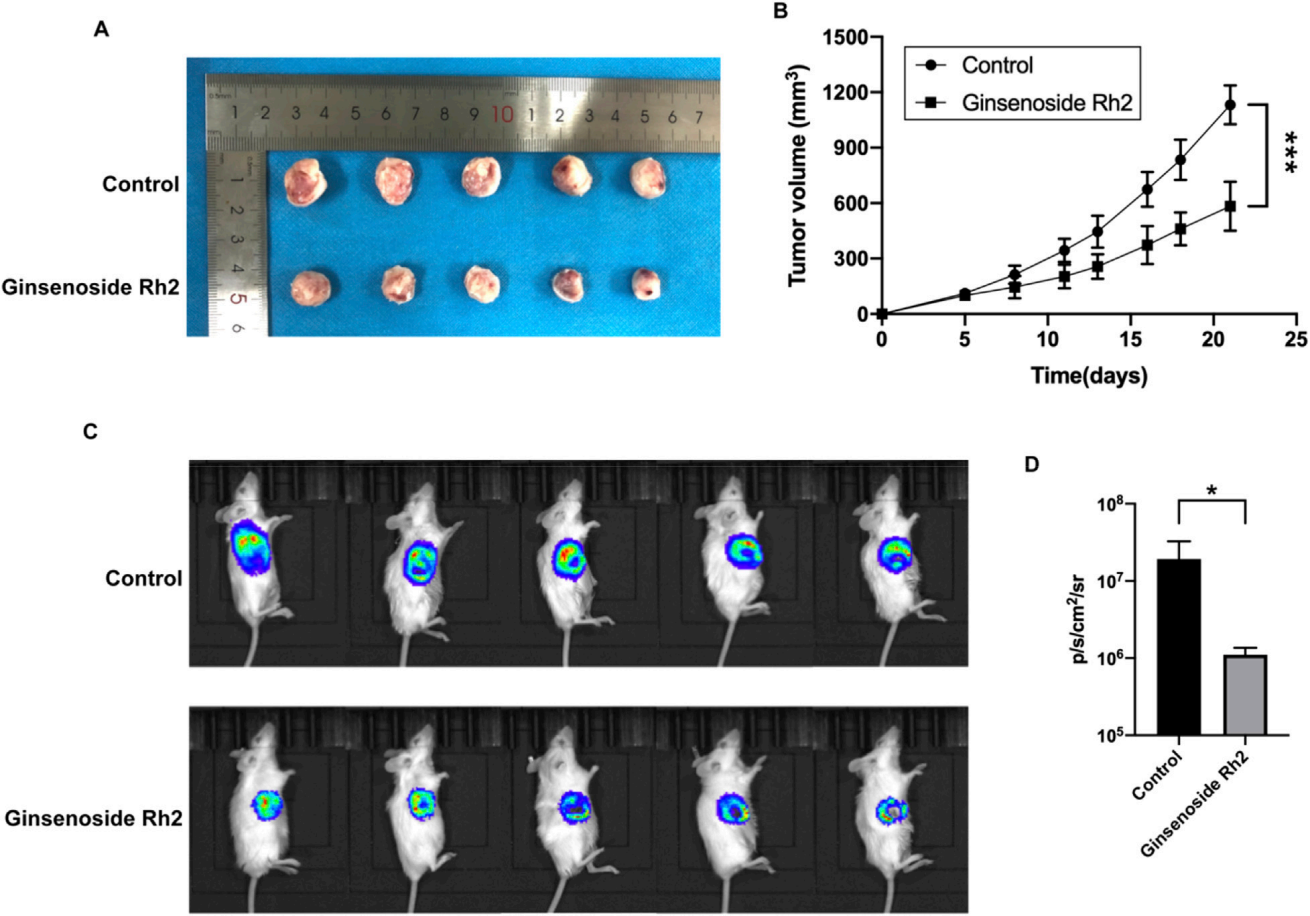
Growth inhibition of TNBC mice treated with ginsenoside Rh2 (50 mg/kg). **(A)** Ginsenoside Rh2 inhibited tumor growth in the TNBC mouse model. **(B)** The growth curves were calculated by tumor volume during 21 days of the treatment with ginsenoside Rh2 by two-way ANOVA test. **(C)** Bioluminescence images of 4T1-luciferase TNBC mouse model after different treatments at the end of the experiment. **(D)** Bioluminescence signals were quantified in the control group and ginsenoside Rh2-treated group by Student's *t*-test. Data are shown as mean ± SD. n ≥ 3; *p < 0.05; ***p < 0.001.

To confirm the inhibitory effect of ginsenoside Rh2 on TNBC tumors *in vivo*, we established a TNBC xenograft mouse model, administered ginsenoside Rh2 treatment, and observed tumor progression. The tumor volume in the ginsenoside Rh2 treatment group was significantly smaller than that in the control group (Figure 3A). The tumor growth curve, as presented in Figure 3B, demonstrated that the ginsenoside Rh2 treatment group exhibited slower tumor growth compared to the control group, with statistically significant differences (*p* < 0.001). These results indicate that ginsenoside Rh2 inhibits tumor growth in TNBC mice. Given the high metastatic potential of TNBC, we investigated the impact of ginsenoside Rh2 on TNBC mice metastasis by assessing bioluminescence. It revealed that the ginsenoside Rh2 group exhibited lower bioluminescent signals than the control group (Figure 3C), and the statistical analysis in Figure 3D indicated that the difference was statistically significant (*p* < 0.05). It suggests that ginsenoside Rh2 can inhibit the growth of TNBC tumors *in vivo*.

### 3.4 Ginsenoside Rh2 inhibits the expression of IL-6, IL-6R, STAT3, Bcl-2, and Bcl-xL proteins in TNBC tissue

**FIGURE 4:**
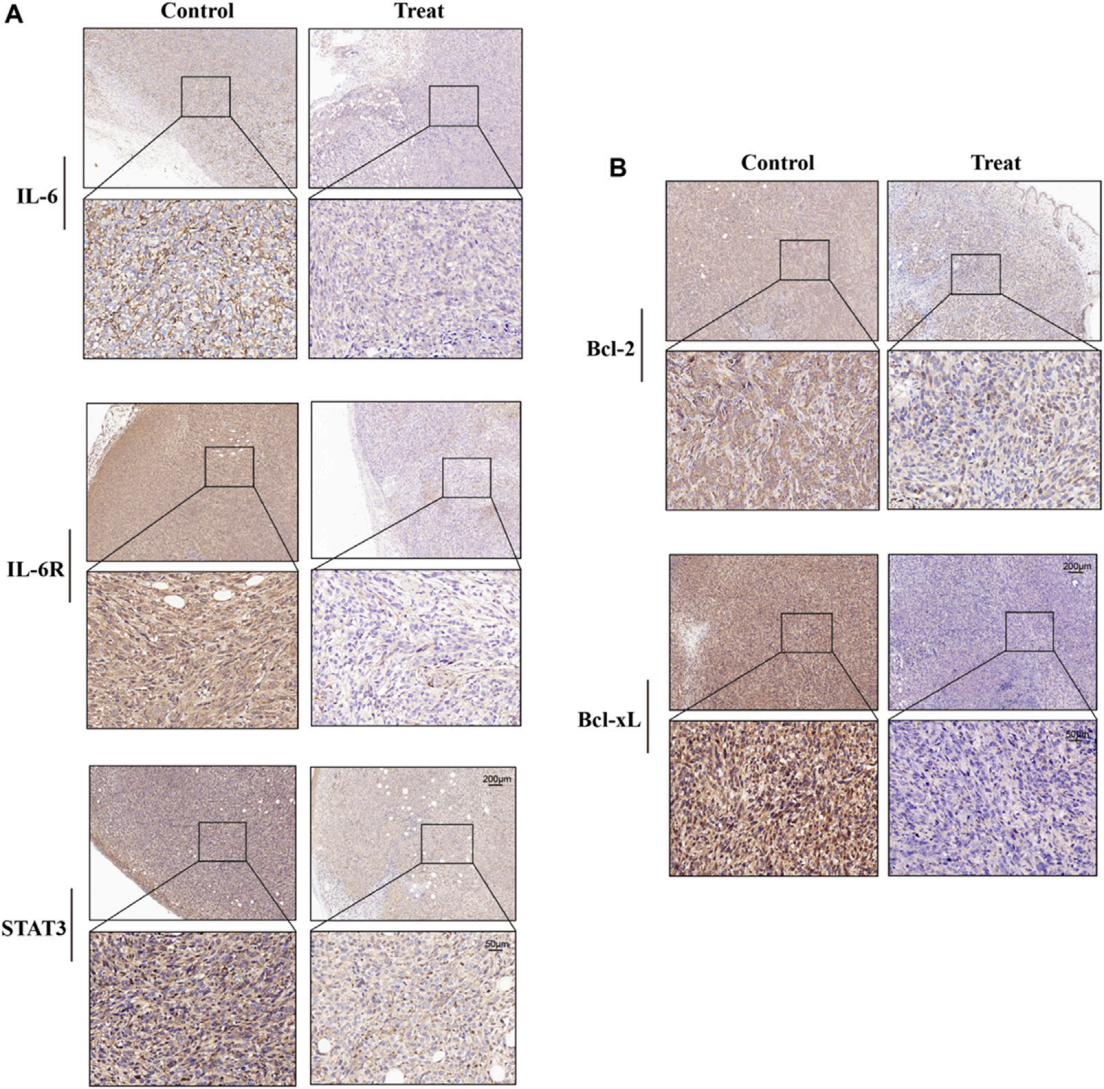
Effects of ginsenoside Rh2 on expression levels of IL-6, IL-6R, STAT3, Bcl-2, and Bcl-xL in tumor tissues of TNBC mice. **(A)** Representative images of IHC staining for IL-6, IL-6R, and STAT3. **(B)** Representative images of Bcl-2 and Bcl-xL IHC staining. Scale bar: 200 μm (upper panel) and 50 μm (lower panel).

Immunohistochemical analysis showed that the expression levels of IL-6, IL-6R, and STAT3 in tumor tissue of mice treated with ginsenoside Rh2 were inhibited (Figure 4A). Moreover, ginsenoside Rh2 also decreased the expression levels of anti-apoptotic proteins Bcl-2 and Bcl-xL (Figure 4B). These results indicate that ginsenoside Rh2 can reduce the activation of the IL-6/JAK2/STAT3 signaling pathway to promote the apoptosis of tumor cells and inhibit the growth of tumors *in vivo*.

### 3.5 Ginsenoside Rh2 inhibits the proliferation, migration, and invasion of TNBC cells

**FIGURE 5:**
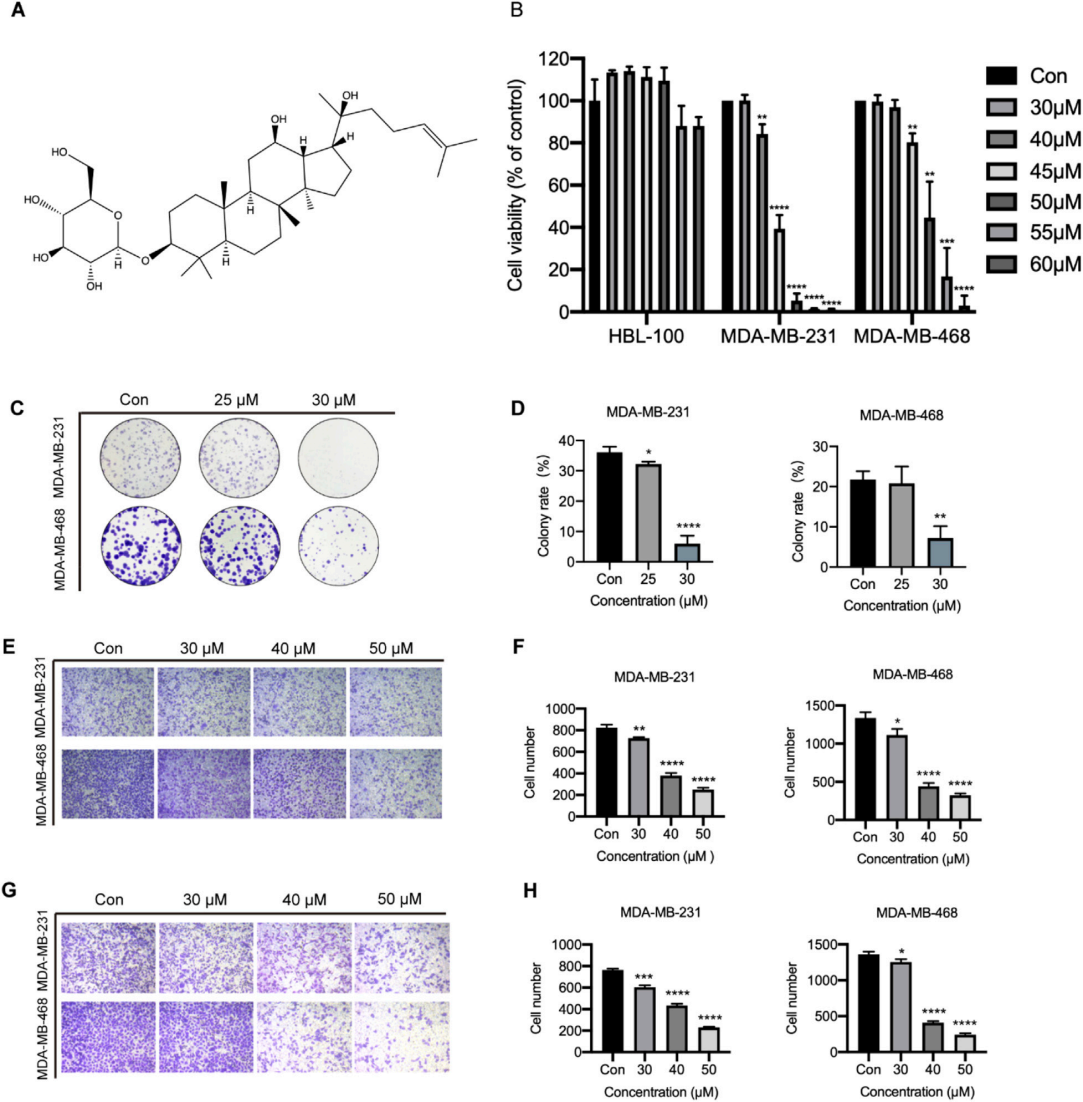
Function of ginsenoside Rh2 on TNBC cell proliferation, migration, and invasion. **(A)** The chemical structure of 20(S)-Rh2. **(B)** Cell viability detection in HBL-100, MDA-MB-231 and MDA-MB-468 with ginsenoside Rh2 at different concentrations for 48 h. **(C)** Cell cloning assay with ginsenoside Rh2 treatment at different concentrations for 10 days **(D)** Quantification of the mean colony rate with TNBC cell lines. **(E)** Cell migration assay after ginsenoside Rh2 treatment at different concentrations for 24 h. **(F)** Quantification of mean migratory cell number with TNBC cell. **(G)** Cell invasion assay after ginsenoside Rh2 treatment at different concentrations for 24 h. **(H)** Quantification of mean invasive cell number with MDA-MB-231 and MDA-MB-468. Data are shown as mean ± SD. n ≥ 3; *p < 0.05; **p < 0.01; ***p < 0.001; ****p < 0.0001 compared with the control group.

Ginsenoside Rh2 has two isomers, denoted as S-type and R-type configurations, and the cytotoxic activity of 20(S)-Rh2 (Figure 5A) is more powerful than 20(R)-Rh2 (Dong et al., 2011). Hence, in our study ginsenoside Rh2 mainly refers to 20(S)-Rh2. The cytotoxic effects of ginsenoside Rh2 on HBL-100, MDA-MB-231, and MDA-MB-468 cells were assessed using the CCK-8 assay. The results indicated that Ginsenoside Rh2 significantly reduced the viability of the TNBC cell lines (MDA-MB-231 and MDA-MB-468) (*p* < 0.05), while exerting no notable cytotoxic effect on normal breast cells (HBL-100) (Figure 5B). The IC50 values of ginsenoside Rh2 for MDA-MB-231 and MDA-MB-468 cell lines are provided in Table 2, offering a comprehensive analysis of its cytotoxicity across different concentrations. The cell cloning assay indicated that ginsenoside Rh2 inhibited TNBC cell lines colony formation (*p* < 0.05, Figures 5C, D). In addition, ginsenoside Rh2 inhibited TNBC cell lines cell migratory capacity (*p* < 0.05, Figures 5E,F) and invasiveness in 3D culture with Matrigel (*p* < 0.05, Figures 5G, H). Ginsenoside Rh2 decreased TNBC cell proliferation, migration, and invasion. We have also included the results of the colony formation assay. These results further demonstrate the ginsenoside Rh2’s inhibitory effects on long-term proliferation in TNBC cells, complementing the findings from the cell viability, migration, and invasion assays. As the concentrations of ginsenoside Rh2 increased, these inhibitory effects markedly strengthened. All these results indicate that ginsenoside Rh2 has an obvious anti-tumor function in TNBC cell lines.

**TABLE 2:** The IC50 values for 48 h treatment of ginsenoside 20(S)-Rh2 in TNBC cell lines.

Cell line	IC_50_ (μM)
MDA-MB-231	43.93 ± 0.50
MDA-MB-468	49.5 ± 2.02

### 3.6 Ginsenoside Rh2 promotes TNBC cell lines apoptosis

**FIGURE 6:**
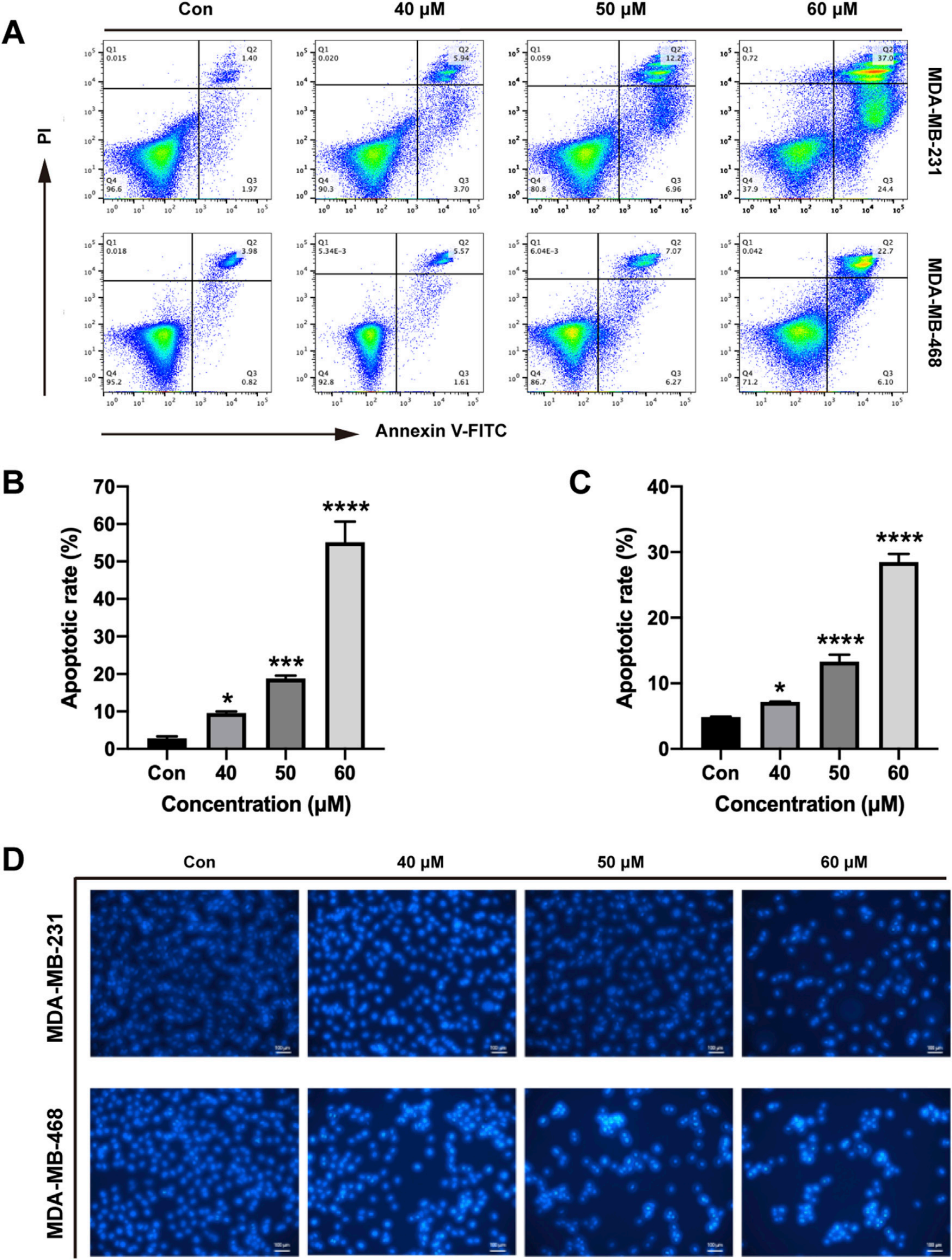
Induction of apoptosis by ginsenoside Rh2 in TNBC cell lines. **(A)** Annexin V/PI dual-staining assay was used to detect apoptosis of MDA-MB-231 and MDA-MB-468 at the indicated concentrations of ginsenoside Rh2. **(B, C)** The apoptotic rate of MDA-MB-231 cell **(B)** and MDA-MB-468 **(C)**. **(D)** Hoechst 33,258 staining was shown in TNBC cell lines (scale bar, 100 μm). Data are shown as mean ± SD. n ≥ 3; *p < 0.05; ***p < 0.001; ****p < 0.0001 compared with the control group.

To explore the function of ginsenoside Rh2 in cell apoptosis, we performed the Hoechst staining experiment and flow cytometry assay. We observed that ginsenoside Rh2 can promote TNBC cell apoptosis in a dose-dependent fashion (*p* < 0.05, Figures 6A–C). Hoechst staining also qualitatively suggested that ginsenoside Rh2 could induce the apoptosis of TNBC (Figure 6D).

### 3.7 Ginsenoside Rh2 inhibits the expression of Bcl-2 and Bcl-xL in TNBC cell lines

**FIGURE 7:**
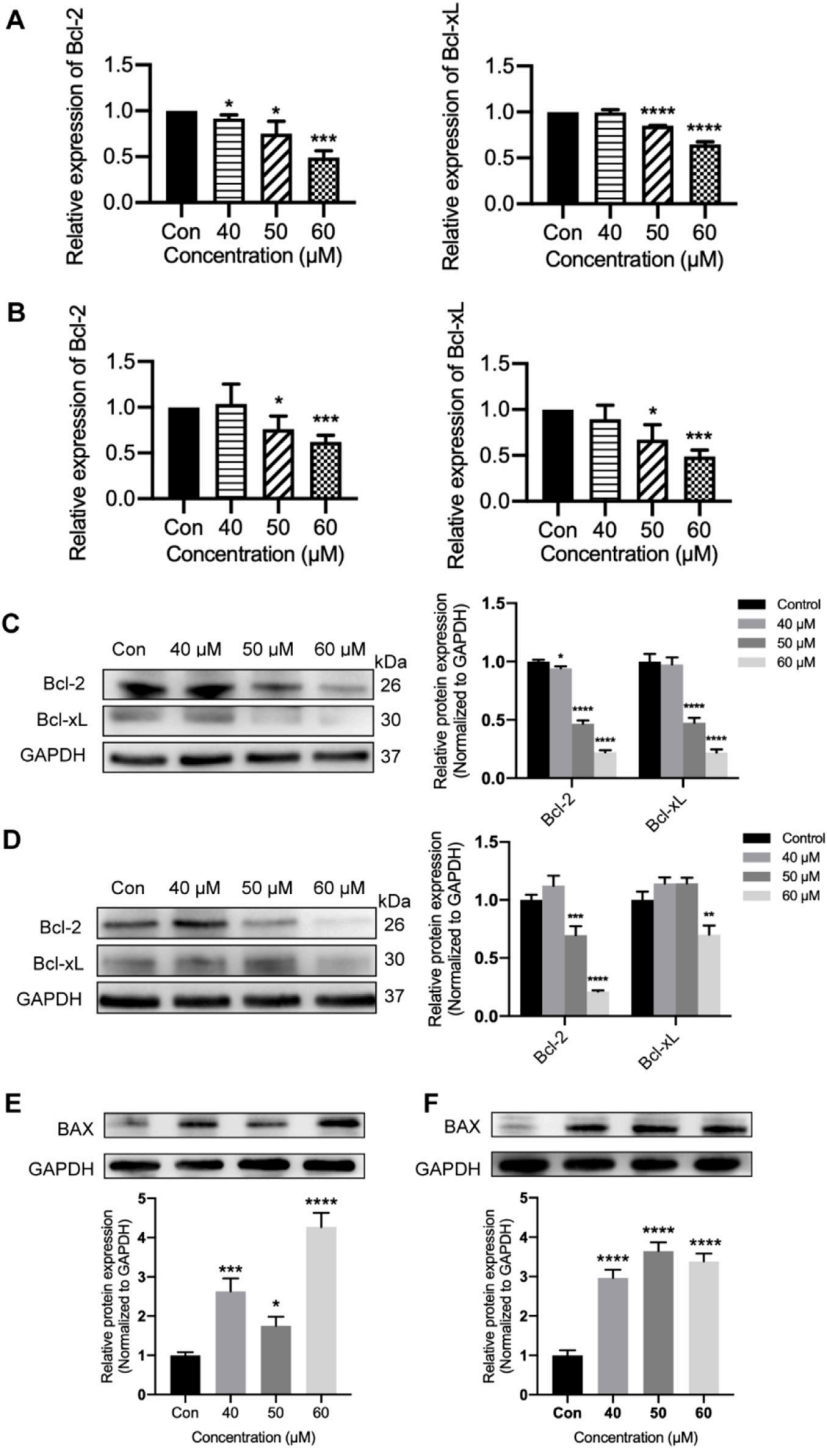
Inhibiting of Bcl-2 and Bcl-xL by ginsenoside Rh2 in TNBC cell lines. **(A, B)** Transcriptional level of Bcl-2 and Bcl-xL in MDA-MB-231 **(A)** and MDA-MB-468 **(B)** with ginsenoside Rh2 at different concentrations. **(C, D)** Cell lysates were analyzed with Bcl-2 and Bcl-xL by Western blotting assay in MDA-MB-231 **(C)** and MDA-MB-468 **(D)** treated with ginsenoside Rh2. **(E, F)** The protein level of pro-apoptotic marker (BAX) was determined by immune-blot in MDA-MB-231 **(E)** and MDA-MB-468 cells **(F)**. Data are shown as mean ± SD. n ≥ 3; *p < 0.05; **p < 0.01; ***p < 0.001; ****p < 0.0001 compared with the control group.

Furthermore, we examined the Bcl-2 and Bcl-xL activities processed with different concentrations of ginsenoside Rh2 to obtain more insights into the pro-apoptosis of ginsenoside Rh2. Ginsenoside Rh2 treatment could inhibit anti-apoptotic genes Bcl-2 and Bcl-xL in terms of the transcription level (*p* < 0.05, Figures 7A, B) and the protein level (*p* < 0.05, Figures 7C, D). In addition, the results from the Western blot analysis, as shown in Figures 7E, F (*p* < 0.05), demonstrate that ginsenoside Rh2 significantly upregulated the expression of the pro-apoptotic gene BAX, indicating its role in promoting apoptosis. These results suggest that ginsenoside Rh2 could play a pro-apoptotic role in TNBC cells.

### 3.8 Ginsenoside Rh2 suppresses the IL-6/JAK2/STAT3 signaling pathway

**FIGURE 8:**
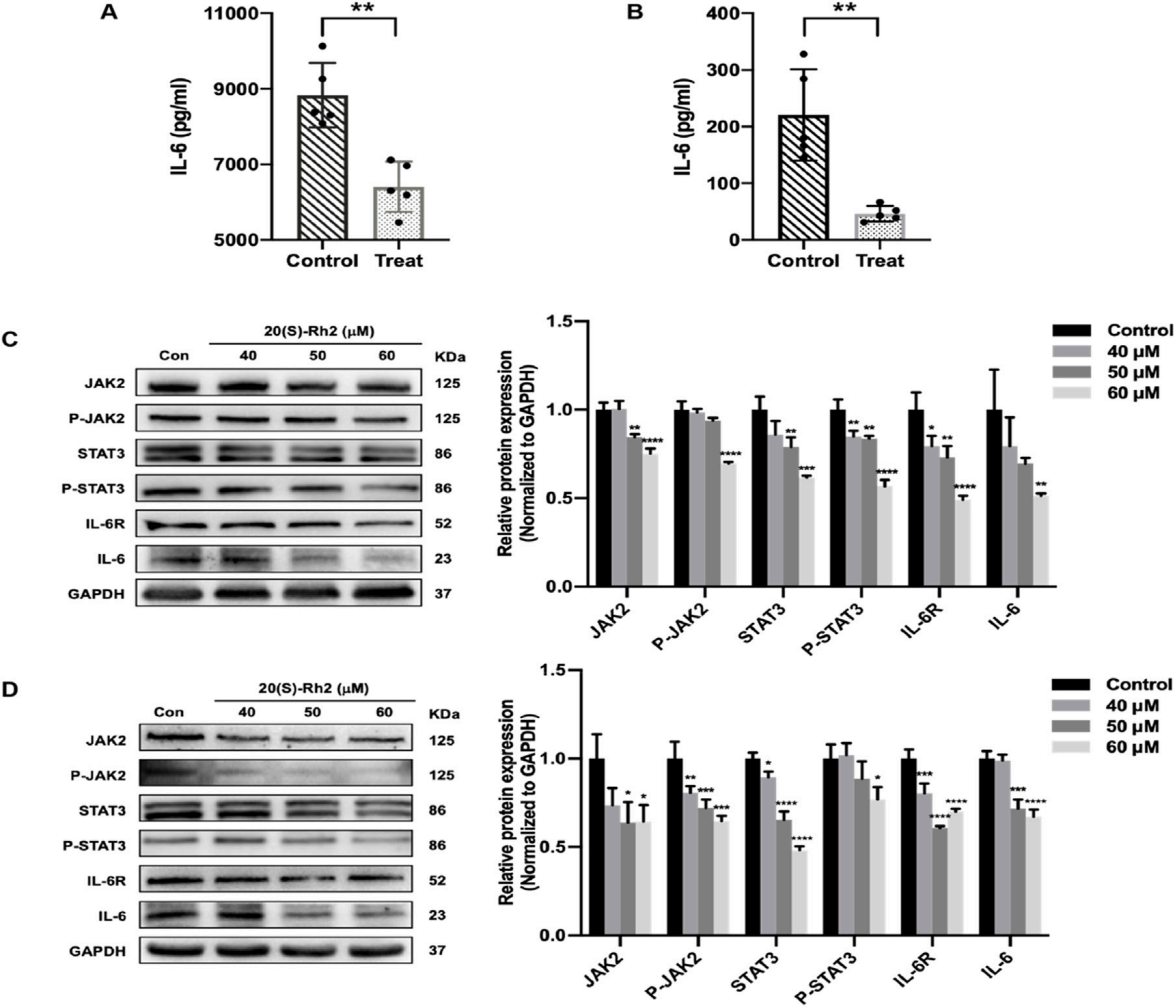
Inhibiting of IL-6/JAK2/STAT3 signaling pathway in TNBC cells treated with ginsenoside Rh2. **(A, B)** Comparison of the expression level of cytokine IL-6 secreted by MDA-MB-231 **(A)** and MDA-MB-468 **(B)** in the control group and ginsenoside Rh2-treated group. **(C, D)** The protein level of the indicated target involved in the IL-6/JAK2/STAT3 signaling pathway in MDA-MB-231 **(C)** and MDA-MB-468 **(D)**. Data are shown as mean ± SD. n ≥ 3; *p < 0.05; **p < 0.01; ***p < 0.001; ****p < 0.0001, in relative to the control group.

To confirm the ability of ginsenoside Rh2 to modulate the IL-6/JAK2/STAT3 signaling pathway within TNBC cells, we examined the expression levels of the cytokine IL-6 and key genes in this pathway, including IL-6, its receptor IL-6R, and STAT3 (P-STAT3), as well as JAK2 (P-JAK2), in MDA-MB-231 and MDA-MB-468 cells. The results revealed that in the ginsenoside Rh2 treatment group, the secretion of the cytokine IL-6 by MDA-MB-231 and MDA-MB-468 cells was significantly inhibited, with a notable difference (*p* < 0.01), suggesting that ginsenoside Rh2 can inhibit the secretion of IL-6 by TNBC cells (Figures 8A, B). Results from the Western blot assay demonstrated that in the ginsenoside Rh2-treated group, the protein expression levels of IL-6 and its receptor IL-6R in MDA-MB-231 and MDA-MB-468 cells were significantly suppressed (*p* < 0.05). Additionally, the protein expression levels of factors on the downstream regulatory signaling pathway of IL-6, JAK2/STAT3, including JAK2, P-JAK2, STAT3, and P-STAT3, were also inhibited (*p* < 0.05, Figures 8C, D). It indicates that ginsenoside Rh2 inhibits the expression of the IL-6/JAK2/STAT3 signaling pathway in TNBC cells.

### 3.9 Ginsenoside Rh2 inhibits protein kinases involving IL-6/JAK2/STAT3 the pathway in TNBC

Aberrant expression of protein kinases in tumor cells is often closely associated with malignant tumor characteristics, including migration, invasion, apoptosis, and chemotherapy resistance. Protein kinases are frequently targeted by anticancer drugs and play a key role in regulating various signaling pathways within tumor cells. PKA-Cα is a cyclic adenosine monophosphate (cAMP)-dependent protein kinase, belonging to the serine/threonine kinase family, which can directly regulate the phosphorylation of JAK2 and STAT3. AMPK-α1 regulated by IL-6 is another serine/threonine kinase closely linked to the IL-6/STAT3 signaling pathway. Therefore, through revealing the molecular mechanisms of how ginsenoside Rh2 regulates the IL-6/JAK2/STAT3 signaling pathway in TNBC cells, this study further explores the effects of ginsenoside Rh2 on the protein kinases PKA-Cα and AMPK-α1.

**FIGURE 9:**
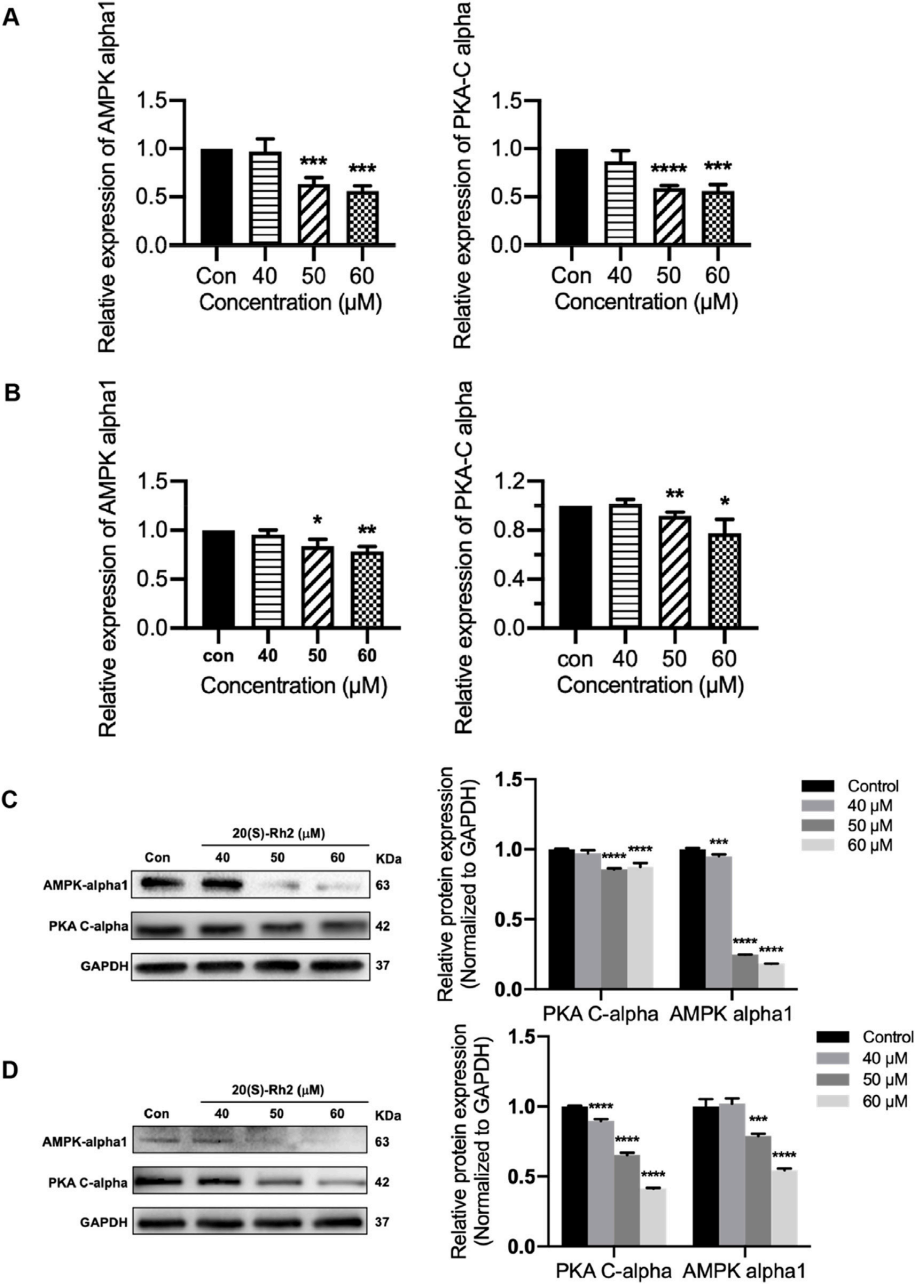
Ginsenoside Rh2 reduces the expression of protein kinase PKA-Cα and AMPK-alpha1. **(A, B)** PKA-Cα and AMPK-alpha1 mRNA levels in MDA-MB-231 **(A)** and MDA-MB-468 **(B)** with ginsenoside Rh2 at different concentrations for 6 h. **(C, D)** Protein level of PKA-Cα and AMPK-alpha1 in MDA-MB-231 **(C)** and MDA-MB-468 **(D)**. Data are shown as mean ± SD. n ≥ 3; *p < 0.05; **p < 0.01; ***p < 0.001; ****p < 0.0001 compared with the control group.

**FIGURE 10:**
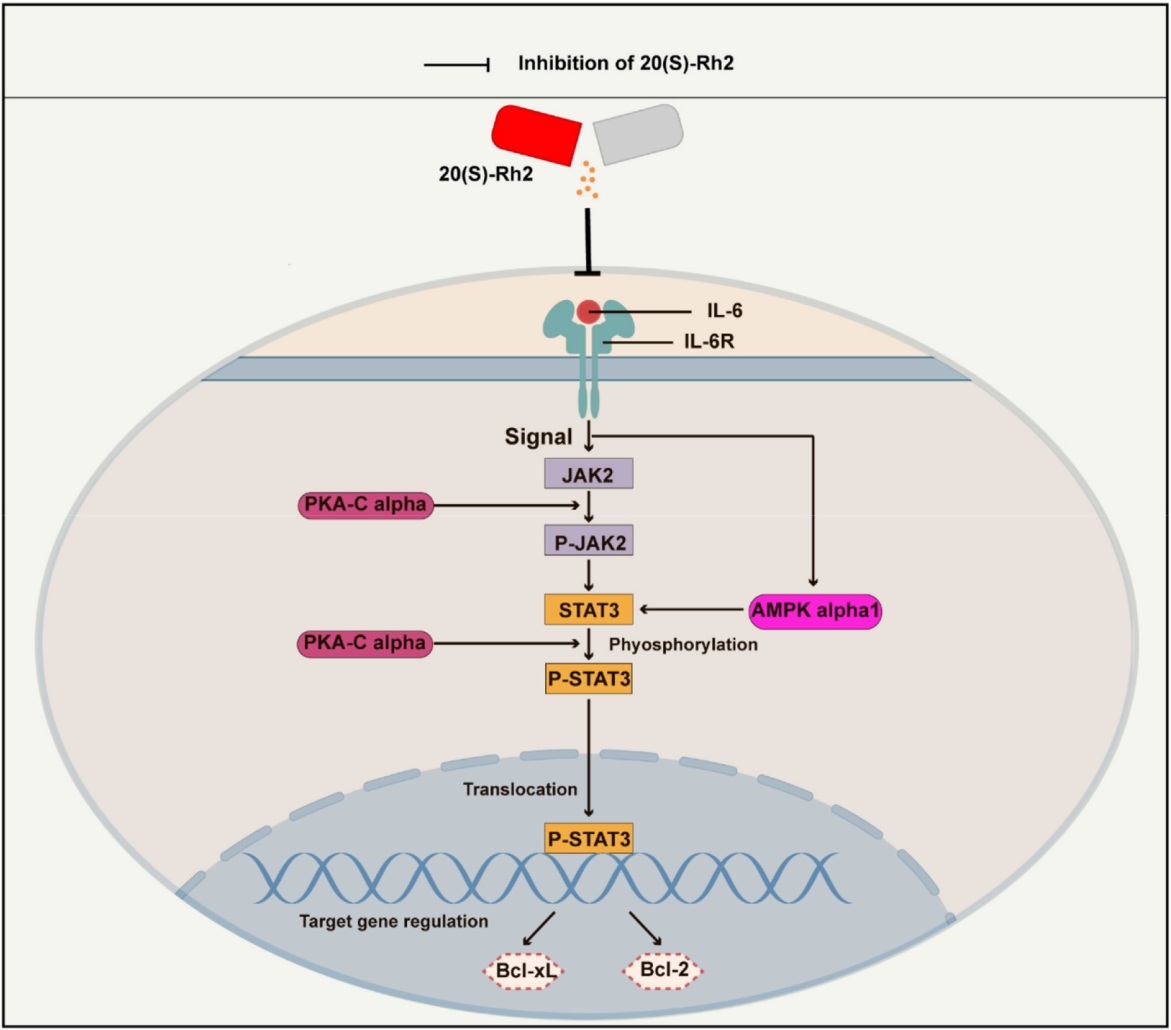
A schematic diagram of ginsenoside Rh2 inhibiting IL-6/JAK2/STAT3 signaling pathway in TNBC cells. Cytokine IL-6 initiates the intracellular IL-6/JAK2/STAT3 signaling pathway by binding to the receptor IL-6R on the surface of the cell membrane. Ginsenoside Rh2 can suppress the secretion of IL-6 by TNBC cells and thus inhibits the activation of IL-6/JAK2/STAT3. Furthermore, ginsenoside Rh2 also can induce the apoptosis of TNBC cells by decreasing the expression of anti-apoptotic proteins Bcl-2 and Bcl-xL. In addition, ginsenoside Rh2 can also inhibit the expression of protein kinases AMPK-alpha1 and PKA-Cα, which directly or indirectly regulates the JAK2/STAT3 pathway.

The results indicated that ginsenoside Rh2 reduced the transcription levels (*p* < 0.05, Figures 9A, B) and protein expression (*p* < 0.001, Figures 9C, D) of PKA-Cα and AMPK-α1 kinases in MDA-MB-231 and MDA-MB-468 cells, suggesting that ginsenoside Rh2 can downregulate the expression of PKA-Cα and AMPK-α1 kinases in TNBC cells. Taken together, ginsenoside Rh2 can suppress the IL-6/JAK2/STAT3 signaling pathway and protein kinases AMPK-α1 and PKA-Cα in TNBC cells (Figure 10). These findings not only elucidate the molecular mechanism by which ginsenoside Rh2 promoted apoptosis in TNBC but also illustrate the process involving multi-target synergism.

## 4 Discussion

Ginsenoside Rh2 has emerged as a potential modulator of the proliferation and apoptosis process in TNBC. In this study, we initially conducted network analysis, revealing that the influence of Ginsenoside Rh2 on the JAK-STAT signaling pathway potentially contributes to the modulation. Further investigation into the underlying biological processes led us to hypothesize that ginsenoside Rh2 exerts its effects on TNBC proliferation and apoptosis via the specific IL-6/JAK2/STAT3 signaling pathway. Subsequent comprehensive experiments were conducted to validate this hypothesis, demonstrating the suppressive effect of ginsenoside Rh2 on the IL-6/JAK2/STAT3 signaling pathway both *in vivo* and *in vitro*.

IL-6, an inflammatory cytokine, is secreted by various cells to maintain internal balance and activates the JAK2/STAT3 signaling transduction pathway by binding to its receptor, IL-6R. JAK2, a non-receptor protein tyrosine kinase, plays a crucial role in the activation of multiple signaling pathways (Chung and Chang, 2003; Jin et al., 2013; Wang et al., 2013; Tanaka et al., 2018; Sopjani et al., 2021). Studies have shown that the expression level of IL-6 is correlated with the prognosis of tumors (Sanguinete et al., 2017; Tanaka et al., 2017). STAT3 is a cytoplasmic protein activated by cytokines and growth factors, participating in gene regulation (Yu et al., 2009; Lee et al., 2019; Mengie Ayele et al., 2022). It is overactivated in breast cancer, promoting tumor cell survival and growth (Bu et al., 2017). STAT3 was found to be strongly expressed in the tumor tissue of TNBC mice in this study, and ginsenoside Rh2 could inhibit its expression (Figure 4). The IL-6/JAK2/STAT3 pathway is involved in various biological processes, including tumor cell proliferation, apoptosis, migration, invasion, and cell cycle progression (Yuan et al., 2020; Zhao et al., 2020; Alaaeldin et al., 2022). Inhibiting this pathway has been shown to suppress tumor cell growth, apoptosis, migration, invasion, and epithelial-mesenchymal transition (Zhu S. et al., 2015; Huynh et al., 2017; Johnson et al., 2018). Targeted inhibitors of the IL-6/JAK2/STAT3 pathway have been used in clinical settings, but their high cost and adverse effects make them less favorable. In contrast, 20(S)-Rh2, as a relatively safe and effective active metabolite of *Panax ginseng*, has not been previously reported for its role in regulating the IL-6/JAK2/STAT3 signaling pathway in TNBC.

PKA is an effector kinase in the cAMP signaling cascade, participating in the regulation of various cellular processes, including cell metabolism, growth, gene expression, and apoptosis (Haidar et al., 2017). PKA-Cα is a catalytic subunit with significant catalytic function within the PKA protein kinase, capable of phosphorylating a range of substrates (Calebiro et al., 2017; Weigand et al., 2021). Studies have shown that PKA-Cα plays a pivotal role in the regulation of the JAK2/STAT3 signaling pathway, exerting important regulatory effects on the phosphorylation of JAK2 and STAT3. PKA inhibitors can suppress STAT3 phosphorylation; cAMP/PKA can enhance the expression of IL-6 mRNA induced by IL-1β and the release of IL-6; PKA activation can inhibit the interferon-induced JAK/STAT signaling pathway (Tanabe et al., 2016; Wang et al., 2017). It has been reported that the activity of the AMPK is closely associated with the IL-6 signaling pathway. Studies have indicated that the activity of AMPK decreases in IL-6 gene knockout mice (Kelly et al., 2004), and the cytokine IL-6 can regulate AMPK activity (Ruderman et al., 2006). Furthermore, research suggests that AMPK-α1 can participate in the process of apoptosis and serves as a cleavage target during apoptosis, specifically cleaved by caspase-3 in the early stages of apoptosis (Cheratta et al., 2022). Additionally, research has shown that AMPK-α1 is closely related to the expression of STAT3 (Zhu Y. P. et al., 2015). In general, protein kinases PKA-Cα and AMPK-α1 play a critical role in regulating the IL-6/STAT3 signaling pathway and the growth and apoptosis of tumor cells. In this research, it was discovered that 20(S)-Rh2 ginsenoside could reduce the expression levels of AMPK-α1 and PKA-Cα in TNBC cells (Figure 9). This finding also corresponded with the network analysis prediction that ginsenoside Rh2 may affect the protein kinase signaling pathway in TNBC cells (Figure 1C).

We have identified the precise targets of Ginsenoside Rh2 and revealed a novel multi-target, synergistic pro-apoptotic mechanism in TNBC. As a natural small molecule inhibitor of the IL-6/JAK2/STAT3 pathway, ginsenoside Rh2 shows great potential as an anti-tumor agent. Future research could focus on investigating the effects of Rh2 on other tumors and various immune cells, as well as exploring different dosage regimens to optimize its therapeutic window and minimize potential side effects. Additionally, the development of targeted small molecule compounds that enhance the selectivity and efficacy of ginsenoside Rh2 could provide further opportunities for clinical application. Our experiments have demonstrated that ginsenoside Rh2 effectively inhibits AMPK-α1 and PKA-Cα expression at both the mRNA and protein levels, suggesting that these kinases may be involved in the regulation of the signaling pathways. However, as our results were primarily focused on the STAT3 pathway, further investigation into the phosphorylation levels of AMPK-α1 and PKA-Cα was not pursued in this study. Given the critical roles that AMPK-α1 and PKA-Cα play in cellular energy regulation and apoptosis, it would be valuable for future studies to explore how Ginsenoside Rh2 affects their phosphorylation status, particularly in relation to the IL-6/JAK2/STAT3 axis. This could provide deeper insights into the broader signaling networks through which Ginsenoside Rh2 exerts its anti-tumor effects.

## 5 Conclusion

In conclusion, the study finds that ginsenoside Rh2 can reduce the growth of TNBC and induce apoptosis, which may be related to the inhibition of IL-6/JAK2/STAT3 signaling pathway and suppression of anti-apoptotic proteins Bcl-2 and Bcl-xL.

## Data Availability

The original contributions presented in the study are publicly available. This data can be found here: https://www.ncbi.nlm.nih.gov/sra/PRJNA1205000.

## Abbreviations

TNBC, Triple-negative breast cancer; IL-6, Cytokine interleukin-6; IL-6R, Cytokine interleukin-6 receptor; JAK2, Janus kinase-2; STAT3, Signal transducers and activator of transcription 3; AMPK-α1, AMP-activated protein kinase alpha1; PKA-Cα, Catalytic alpha of cAMP-dependent protein kinase PKA-Cα; RT-PCR, Real-time polymerase chain reaction; GO, Gene Ontology; KEGG, Kyoto Encyclopedia of Genes and Genomes; BP, Biological process; CC, Cellular component; MF, Molecular function.
