# Supplementary material for: Ginsenoside Rh2 regulates triple-negative breast cancer proliferation and apoptosis via the IL-6/JAK2/STAT3 pathway
Source: Front Pharmacol. 2025 Jan 8;15:1483896. doi: 10.3389/fphar.2024.1483896 (PMC11751231; doi:10.3389/fphar.2024.1483896)
Supplement: Supplementary file 1 [file Table1.DOCX]

**Clone formation experiment**

2 mL complete medium including various concentrations of ginsenoside Rh2 mixed with 500 cells was added to each well of a 6-well plate. The 6-well plate was placed in the incubator for further cultivation. After 10 days, paraformaldehyde (biosharp, Beijing, China) was fixed overnight and 0.1% crystal violet (meilunbio, Dalian, China) was dyed for 1 h. The number of clones was assessed by photographing after water washing.

The Cell cloning assay Ginsenoside Rh2 inhibited TNBC cell lines colony formation ( Supplementary Figure. 1)


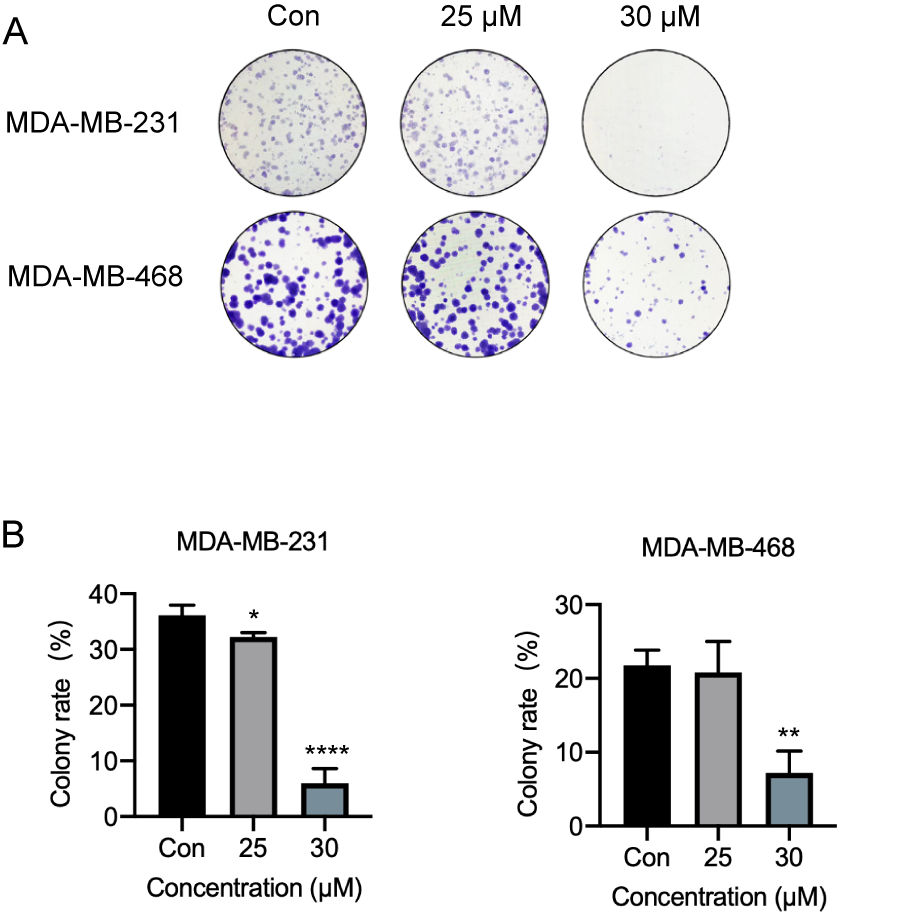


**Figure 1. Determination of the cell cloning viability with ginsenoside Rh2 treatment in TNBC cell lines.** (A) Cell cloning assay with ginsenoside Rh2 treatment at different concentrations for 10 days. (B) Quantification of the mean colony rate with MDA-MB-231 and MDA-MB-468. Data are shown as mean ± SD. n ≥ 3; **P* < 0.05; ***P* < 0.01; *****P* < 0.0001; Student's *t*-test.
